# Impact of preocular and ocular circulatory dynamics on the vascular density of retinal capillary plexuses and choriocapillaris

**DOI:** 10.1007/s00417-024-06544-4

**Published:** 2024-07-31

**Authors:** Rodolphe Vallée, Dilsah Körpe, Jean-Noël Vallée, Georgios N. Tsiropoulos, Daniela Gallo Castro, Irmela Mantel, Constantin J. Pournaras, Aude Ambresin

**Affiliations:** 1 Present Address: Diagnostic and Functional Neuroradiology and Brain Stimulation Department - Clinical Investigation Center 1423, 15-20 National Vision Hospital, University of Paris-Saclay - UVSQ, 28, Rue de Charenton, Paris, 75012 France; 2 Laboratory of Mathematics and Applications (LMA) CNRS 7348, LRCOM i3M -DACTIM-MIS (Data Analysis and Computations Through Imaging Modeling Mathematics, University of Poitiers, Poitiers, France; 3 Swiss Visio Montchoisi, Lausanne, Switzerland; 4 Faculty of Biology and Medicine, University of Lausanne (UNIL), Lausanne, Switzerland; 5 RétinElysée, Ophthalmology Center, Lausanne, Switzerland; 6 Department of Health Sciences, Medical School, Aristotle University of Thessaloniki (A.U.Th), Thessaloniki, Greece; 7 Department of Ophthalmology, Jules Gonin Eye Hospital, Fondation Asile Des Aveugles, Lausanne, Switzerland; 8 Hirslanden Clinique La Colline, Geneva, Switzerland

**Keywords:** Choriocapillaris, Fluorescein angiography, Indocyanine angiography, OCT angiography, Retinal capillary, Retinal imaging

## Abstract

**Purpose:**

To highlight the influence of preocular and ocular vascular circulatory dynamics on the vascular density (VD) of retinal capillary plexuses (RCPs) and choriocapillaris (CC) in patients with and without cardiovascular risk (CVR) factors.

**Methods:**

A retrospective observational study in patients with and without CVR factors (type 1 and 2 diabetes, arterial hypertension, and hypercholesterolemia). Fluorescein (FA) and indocyanine (ICGA) angiography circulatory times were arterial time (FA_AT_), start (FA_startLF_) and end (FA_endLF_) of laminar flow, and arterial time (ICGA_AT_), respectively. OCT angiography VDs were superficial (VD_SCP_) and deep (VD_DCP_) RCPs and CC (VD_CC_) VDs. Correlation and regression analysis were performed after adjusting for confounding factors.

**Results:**

177 eyes of 177 patients (mean age: 65.2 ± 15.9 years, n = 92 with and 85 without CVR) were included. VD_SCP_ and VD_DCP_ were significantly inversely correlated with FA_AT_, FA_startLF_ and FA_endLF_ likewise VD_CC_ with ICGA_AT_. Correlations were stronger in patients without CVR than with CVR. CVR, FA_AT_, FA_startLF_ and FA_endLF_ were more strongly correlated with VD_DCP_ than VD_SCP_. FA_AT_, FA_startLF_ and FA_endLF_ significantly impacted VD_SCP_ and VD_DCP_, likewise ICGA_AT_ impacted VD_DCP_. VD_DCP_ was most strongly impacted by FA_AT_ and FA_startLF_.

**Conclusion:**

Ocular and pre-ocular circulatory dynamics significantly impacted RCPs and CC VDs, especially deep RCP.

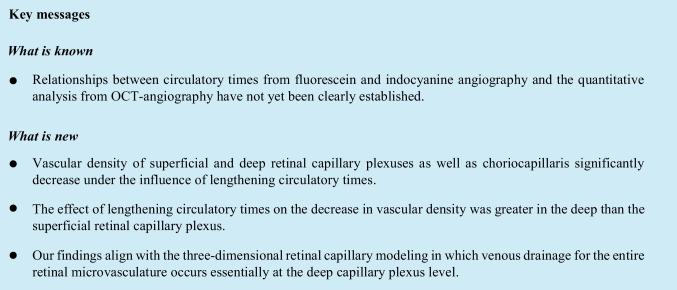

## Introduction

The retina is vascularized by two distinct vascular network systems without anatomical connections under physiological conditions. The retinal capillary network arises from the central retinal artery and ensures the direct vascularization of the inner retina. The retinal circulation is a terminal arterial system devoid of anastomoses. The choroidal network arises from the posterior ciliary arteries and ensures the vascularization of outer retina and of the photoreceptors indirectly, since there are no capillaries in the outer retina.

Retinal blood flow plays a crucial role in transporting metabolic substances and oxygen to the retina through the internal blood-retinal barrier, which selectively seals [1]. The maintenance of the inner retina's structure and function relies on the finely tuned regulation of retinal flow. This flow remains constant and self-regulated through mechanisms involving a myogenic aspect, predominantly influenced by the vascular endothelium, and a metabolic aspect associated with the metabolic activities of neurons and glial cells [1]. Retinal ischemic microangiopathies linked to cardiovascular risk factors, such as type 1 and 2 diabetes, arterial hypertension, and hypercholesterolemia, induce during their evolution alterations in neuronal function and retinal endothelial cell structure, that disrupt the interactions of these two metabolic pathways and lead to dysregulation of retinal flow. Damages to retinal capillaries result in retinal ischemia and/or alterations of the blood-retinal barrier [2].

Dye angiography and optical coherence tomography angiography (OCT angiography) enable the investigation of parameters inherent to the hemodynamics and the vascular architecture of the human eye. Fluorescein (FA) and indocyanine (ICGA) angiographies provide dynamic two-dimensional, not depth resolved visualization of retinal and choroidal vascular circulation with a wide field of view, however limiting assessment of deeper retinal capillary layers [3, 4]. Dye angiography is invasive and time-consuming (up to 30 min), incurring significant personnel costs. Adverse effects of the dye include nausea, vomiting, urticaria, syncope, and potentially severe complications affecting the respiratory or cardiac systems [5]. Indocyanine green dye is contraindicated in pregnancy and kidney disease. Despite these limitations, dye-based angiography remains valuable, particularly for detecting vessel leakage or microaneurysms.

OCT angiography is a recent modality for non-invasive, depth resolved, three-dimensional imaging of the retinal and choroidal vasculature. It detects blood flow down to the capillary level by analyzing decorrelation in signal between the sequential cross-sectional images (B-Scans) captured precisely at the same location [6, 7]. Unlike traditional methods involving dye intravenous injection, OCT angiography utilizes the motion of flowing blood cells as intrinsic contrast, rendering it more convenient for routine clinical use [4, 8, 9]. Simultaneous analysis of structural and angiographic data allows for precise localization of vessels on cross-sectional or en-face images, producing volumetric structural and blood flow angiography images within seconds with high spatial resolution. This facilitates the visualization of distinct layers in the retina, enabling automated segmentation to specific depths [6], and provide quantitative (vascular density and perfusion metrics) and qualitative analysis in various retinal vascular disorders [9, 10]. Nevertheless, this imaging modality has some limitations, such as its inability to offer precise quantitative information on blood flow rate and velocity[10] despite the development of various techniques [7, 11]. It offers a restricted field of view [12], typically ranging from 2 × 2 mm to 12 × 12 mm depending on the device, albeit at the cost of resolution. The use of montage techniques enables a wider field of view while preserving improved resolution [12]. However, OCT angiography fails to visualize vessel leakage or provide data on vessel permeability. Moreover, this technique is susceptible to artifacts such as blinks, movements, and ghosting of vessels. It is limited by its capability to detect slowest detectable blood flow, potentially missing the detection of extremely slow (e.g., microaneurysms or fibrotic choroidal neovascularization) or excessively rapid flows. The detection of flow is constrained within a specific dynamic range determined by the time interval between sequential OCT B-scans.

Given that OCT angiography remains a relatively recent technology, the potential association relationships between circulatory times from dye-based angiography and the quantitative analysis from OCT-angiography have not yet been clearly established. Therefore, the primary objective of this study was to explore the extent to which circulatory dynamics in the preocular and ocular regions, as observed by dye-based angiography, impact the vascular density of retinal capillary plexuses and choriocapillaris measured by OCT angiography in patients with and without cardiovascular risk factors.

## Methods

### Study cohort

A single-center retrospective observational study, approved by the Swiss Ethics Committee (approval number: 2019–01615), was carried out from 2019 to 2022 in accordance with the ethical principles of the Declaration of Helsinki. The patient cohort was selected from the RétinElysée database in Lausanne, Switzerland. Inclusion criteria comprised the requirement for both dye and OCT angiographies performed on the same day, an OCT angiography quality index greater than 6, automatic segmentation correction performed by the OCT angiography device software, and patients with a refractive error within ± 3.0 diopters. Exclusion criteria encompassed patients with wet age-related macular degeneration, epiretinal membrane causing alterations in retinal vascular architecture, significant drusen causing a masking effect on the OCT angiography choroidal slab and rendering it unanalyzable, and occlusion or stenosis exceeding 50% of the internal carotid artery. The study cohort was further stratified into two groups based on cardiovascular risk (CVR) factors. The “CVR” group included patients diagnosed with at least one of the microangiopathies like type 1 and 2 diabetes, arterial hypertension, and/or hypercholesterolemia. The "no_CVR" group consisted of patients without any microangiopathies.

### Image processing and variable acquisitions

The demographics (age, gender), cardiovascular (blood pressure measured on the day of angiographic examinations, mean blood pressure ${\text{BP}}_{\text{mean}}=\frac{\text{systolic pressure }+2 \times \text{ disatolic pressure}}{3}$, heart rate during angiography), and ophthalmological data (best corrected visual acuity, refractive error, intraocular pressure (mmHg), circulatory times from fluorescein and indocyanine angiographies, capillary vascular density measured using OCT angiography) as well as patients' medical histories, were collected from the RétinElysée database.

Retinal and choroidal circulatory times (arterial time: FA_AT_, start of laminar flow: FA_startLF_, end of laminar flow: FA_endLF_), and (arterial time: ICGA_AT_) were measured from dynamic film data of early phases of fluorescein angiography (FA, Spectralis OCT, Heidelberg Inc®) and indocyanine green angiography (ICGA, Spectralis OCT, Heidelberg Inc®), respectively [13]. FA_AT_ and ICGA_AT_ were the times to onset of retinal and choroidal arteriolar filling from fluorescein and indocyanine angiography, respectively, FA_startLF_, the time to onset of early laminar flow in the veins objectified by the onset of the dye lining the venous wall leaving an axial hypo fluorescent strip, and FA_endLF_, the time to onset of complete venous filling. These times were manually recorded post-dye injection.

**Fig. 1:**
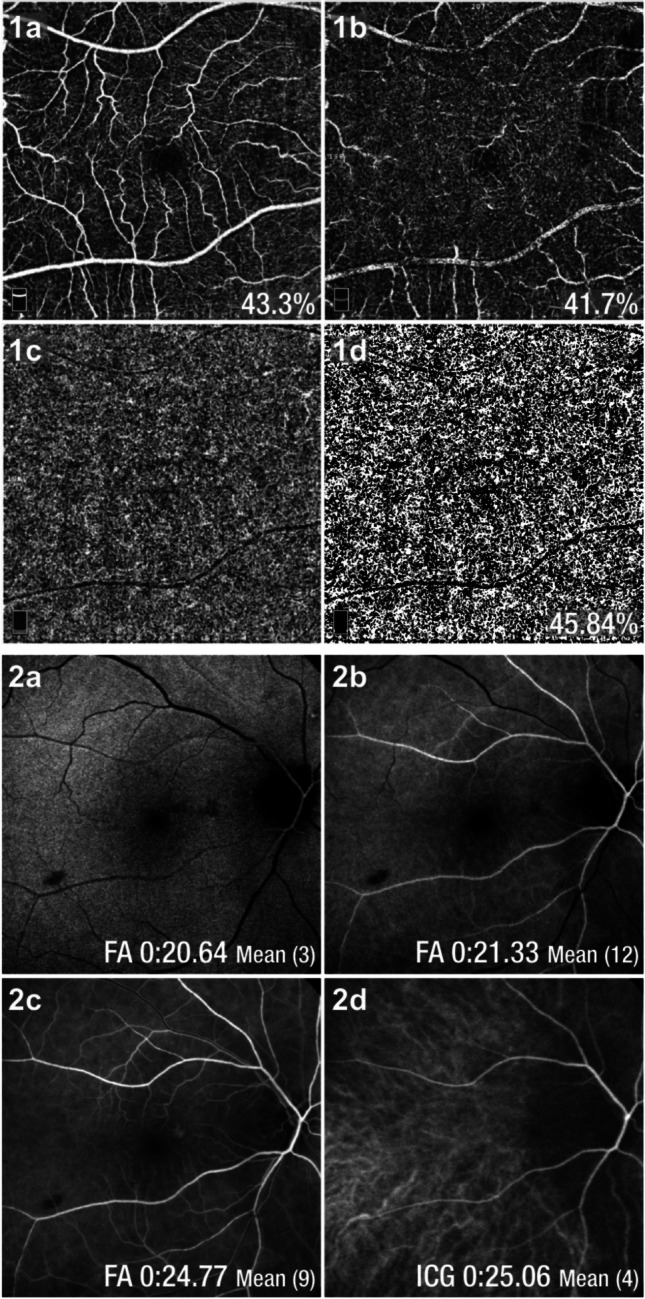
(1) OCT angiography and vascular density: 1a) Superficial capillary plexus, 1b) deep capillary plexus, 1c) choriocapillaris, 1d) choriocapillaris binarized image (FIJI software). (2) Fluorescein angiography and circulatory times: 2a) arterial time, 2b) early laminar flow and 2c) late laminar flow, and 2d) indocyanine angiography and arterial time corresponding to the arrival of dye in the choroidal plexus

OCT angiography (6.0 × 6.0 mm scan size; Optovue, Angiovue®, RTVue XrPAR, version 2017.1.0.150) data were utilized to extract measurements of vascular density of superficial and deep capillary plexuses (VD_SCP_ and VD_DCP_, respectively) of the fovea and of the whole image. Raw choroidal slabs obtained using OCT angiography were exported to Fiji software, a derivative of ImageJ [14] for binarization and subsequent calculation of choriocapillaris vascular density (VD_CC_) (Fig. 1) [15]. The binarization process employed intensity thresholding using Otsu's method, [15, 16] which assumes a bimodal distribution of pixel classes to determine an optimal threshold for separating blood flow signal (white pixels) from regions without blood flow (black pixels) [14]. Binarized images were interpreted and analyzed using the Vessel Analysis plugin in Fiji [17], enabling the computation of choriocapillaris vascular density expressed as a percentage. This measurement was derived by calculating the total vessel-occupied pixels divided by the entire image's pixel count [15].

### Statistical analyses

Statistical analyses were carried out on the overall patient population and on the two distinct patient groups “no_CVR” and “CVR” and performed after adjusting for age, gender and angiogram quality index covariates. Continuous variables such as age, FA_AT_, FA_startLF_, FA_endLF_, ICGA_AT_, VD_SCP_, VD_DCP_ and VD_CC_ were expressed as means and standard deviations (± SD). Categorical variables such as gender (female/male), CVR (yes/no) were expressed as frequencies and percentages (%). The required sample size computed was n = 75 to test the influence of continuous variables on another continuous variable in a simple regression analysis with a type I error of 0.05, a type II error of 0.10, a power of 90%, a coefficient of determination R2 conservatively estimated at 0.2 in the absence of information from the literature, and an expected effect size conservatively estimated at 0.33 in the range between a small effect size of 0.2 and a medium effect size of 0.5. After adding 10% of the calculated sample size to compensate for any study issues, the minimum sample size needed for each group was n = 84.

Statistical relationships of association between the two variables vascular density and circulatory time were assessed using Spearman’s correlation. Strength and direction of association were measured by the Spearman’s correlation coefficient $\rho$. Regression models were implemented to assess the preocular and ocular circulatory dynamics effects on vascular density of retinal capillary plexuses and choriocapillaris. The goodness-of-fit measure of regression models was assessed by the R-squared (R^2^) metric calculation, which represents the percentage of variance of the vascular density variables that can be influenced by the circulation time variables included in the model. Comparisons of the strength of correlation coefficients Rho (ρ) and determination coefficients R^2^ were performed using Z statistic tests.

Data analyses were performed on a full analysis set using multivariate regression imputation of missing data. A two-tailed, False Discovery Rate (FDR) adjusted p-value of less than 0.05 was considered statistically significant (SAS software, version 9.4; SAS Institute, Carry, NC).

## Results

### Demographic synopsis

177 eyes of 177 patients with various eye pathologies (mean age of 65.2 ± 15.9 years) were included in the full analysis set based on the inclusion and exclusion criteria. Ocular pathologies included dry age-related macular degeneration, diabetes without or with minimal to moderate nonproliferative diabetic retinopathy, macroaneurysms, macular epitheliopathy, arterial and venous vascular occlusion, pachychoroid, and central serous chorioretinopathy. “CVR” group included 92 eyes from 92 patients (51.98%; mean age: 69.2 ± 13.7 years; M/F ratio: 0.96), and “no_CVR” group, 85 eyes from 85 patients (48.02%; mean age: 60.7 ± 17.1 years; M/F ratio: 1.07). 18 patients suffered from diabetes, 68 from hypertension and 26 from hypercholesterolemia. 71, 16 and 3 patients had 1, 2 and 3 cardiovascular risk factors, respectively, and 2 without specification on the type of microangiopathy. Diabetic patients did not have diabetic retinopathy or had minimal to moderate nonproliferative diabetic retinopathy. Among patients with hypertension, 37 had high blood pressure with a mean arterial pressure of 117,76 ± 7.07 mmHg. 11 eyes of 11 patients had some missing data. Data of circulatory times and vascular densities are summarized (mean ± SD) in Table 1.

**Table 1:** Demographic synopsis, measurements (mean ± SD) of circulatory times and vascular densities of capillary plexuses, and correlations between vascular densities of capillary plexuses and circulatory times, in patients with and without cardiovascular risk factors

	Without CVR factors (n = 85, 48.02%)				With CVR factors (n = 92, 51,98%)												Cohort (n = 177)			
					All				Diabetes (n = 18)				Other microangiopathies (n = 74)							
Demographic synopsis																				
Age	60.7 ± 17.1				69.2 ± 13.7												65.2 ± 15.9			
Gender	Female *n* = 41 (23.16%)				Female *n* = 47 (26.55%)												Female *n* = 88 (49.72%)			
	Male *n* = 44 (24.86%)				Male *n* = 45 (25.42%)												Male *n* = 89 (50.28%)			
Measurements (Mean ± SD)																				
Fluorescein Angiography: circulatory times (s)																				
FA_AT_	18.59 ± 1.93				19.35 ± 1.68				19.48 ± 1.55				18.84 ± 2.09				18.99 ± 1.84			
FA_startLF_	21.40 ± 1.86				22.10 ± 1.64				22.21 ± 1.52				21.64 ± 2.05				21.76 ± 1.78			
FA_endLF_	28.89 ± 2.15				29.50 ± 2.01				29.58 ± 1.88				29.16 ± 2.49				29.20 ± 2.09			
Indocyanine green angiography: circulatory times (s)																				
ICGA_AT_	17.86 ± 1.90				18.62 ± 1.65				18.75 ± 1.52				18.10 ± 2.04				18.26 ± 1.81			
Optical coherence tomography angiography: vessel density whole image % 6.0																				
VD_SCP_	48.38 ± 2.51				47.12 ± 2.02				46.88 ± 1.96				48.11 ± 2.00				47.73 ± 2.35			
VD_DCP_	48.21 ± 2.76				46.89 ± 2.25				46.64 ± 2.12				47.89 ± 2.51				47.52 ± 2.59			
Quality index	8.5 ± 0.7				8.1 ± 0.8				8.1 ± 0.78				8.1 ± 0.83				8.3 ± 0.8			
VD_CC_	47.73 ± 3.10				46.22 ± 2.54				45.91 ± 2.58				47.50 ± 1.92				46.95 ± 2.91			
Spearman’s $\uprho$ correlations																				
Circulatory times (CTs)	Vascular densities				Vascular densities				Vascular densities				Vascular densities				Vascular densities			
	VD_SCP_		VD_DCP_		VD_SCP_		VD_DCP_		VD_SCP_		VD_DCP_		VD_SCP_		VD_DCP_		VD_SCP_		VD_DCP_	
	ρ	p*	ρ	p*	ρ	p*	ρ	p*	ρ	p*	ρ	p*	ρ	p*	ρ	p*	ρ	p**	ρ	p**
FA_AT_	-0.84	< *0.001*	-0.93	< *0.001*	-0.75	< *0.001*	-0.90	< *0.001*	-0.91	< *0.001*	-0.97	< *0.001*	-0.73	< *0.001*	-0.89	< *0.001*	-0.71	< *0.001*	-0.81	< *0.001*
FA_startLF_	-0.79	< *0.001*	-0.90	< *0.001*	-0.70	< *0.001*	-0.86	< *0.001*	-0.90	< *0.001*	-0.96	< *0.001*	-0.70	< *0.001*	-0.86	< *0.001*	-0.62	< *0.001*	-0.73	< *0.001*
FA_endLF_	-0.53	< *0.001*	-0.69	< *0.001*	-0.53	< *0.001*	-0.73	< *0.001*	-0.83	< *0.001*	-0.91	< *0.001*	-0.48	< *0.001*	-0.68	< *0.001*	-0.46	< *0.001*	-0.62	< *0.001*
	VD_CC_				VD_CC_				VD_CC_				VD_CC_				VD_CC_			
	ρ		p*		ρ		p*		ρ		p*		ρ		p*		ρ		P**	
ICGA_AT_	-0. 66		< *0.001*		-0.44		< *0.001*		-0.66		< *0.001*		-0.47		< *0.001*		-0.58		< *0.001*	

*CVR* cardiovascular risk, * p-value after adjusting for age, gender, scan quality index, ** p-value after adjusting for age, gender, scan quality index, CVR factors, $\rho$ correlation coefficient, *FA* fluorescein angiography, *ICGA* indocyanine angiography, *VD* vascular density, *SCP* superficial retinal capillary plexus, *DCP* deep retinal capillary plexus, *CC* choriocapillaris, *AT* arterial time, *startLF* start of laminar flow, *endLF* end of laminar flow

### Correlation analyses

In the overall population and in the “CVR" and “no_CVR” patient groups, vascular densities VD_SCP_ and VD_DCP_ from OCT angiography were significantly inversely correlated with circulatory times FA_AT_, FA_startLF_, and FA_endLF_ from fluorescein angiography likewise vascular density VD_CC_ from OCT angiography was significantly inversely correlated with circulatory time ICGA_AT_ from indocyanine angiography ($\rho$ <*0; p* < 0.001, respectively), Table 1.

Comparisons of correlation coefficients showed that vascular densities were significantly more strongly correlated with the circulatory times FA_AT_ and FA_startLF_ than with the circulatory times FA_endLF_ and ICGA_AT_ (*p* < 0.05 respectively). There was no significant difference in strength of the correlation coefficients between vascular densities and circulatory times FA_AT_ and FA_startLF_ (p > 0.05 respectively, Table 2.

**Table 2:** Comparisons of the strength of correlation coefficients of the correlations between vascular densities of capillary plexuses and circulatory times, according to A) circulatory times, B) cardiovascular risk status, C) superficial and deep retinal capillary plexuses and D) diabetic microangiopathy compared to other microangiopathies, in patients with and without cardiovascular risk factors

Comparison of correlation coefficients $\rho$	A) ρ(VD, CT_j_) vs ρ(VD, CT_j’_)											
Circulatory times (CTs)	Without CVR factors						With CVR factors					
	Vascular densities						Vascular densities					
	VD_SCP_			VD_DCP_			VD_SCP_			VD_DCP_		
	Z	p*		Z	p*		Z	p*		Z		p*
_j_ = FA_AT_ and _j’_ = FA_startLF_	0.95	0.344		1.35	0.179		0.78	0.437		1.15		0.251
_j_ = FA_startLF_ and _j’_ = FA_endLF_	3.00	0.003		3.77	< 0.001		1.85	0.065		2.54		0.011
	VD_CC_						VD_CC_					
_j_ = FA_startLF_ and _j’_ = ICGA_AT_	1.77	0.077		4.20	< 0.001		2.60	0.009		5.49		< 0.001
Comparison of correlation coefficients $\rho$	B) ρ(VD, CT)_no_CVR_ vs ρ(VD, CT)_CVR_				C) ρ(VD_DCP_, CT) vs ρ(VD_SCP_, CT)				D) ρ(VD, CT)_CVR-diabetes_ vs ρ(VD, CT)_CVR-others_			
Circulatory times (CTs)	Vascular densities				CVR status				Vascular densities			
	VD_SCP_		VD_DCP_		Without CVR factors		With CVR factors		VD_SCP_		VD_DCP_	
	Z	p*	Z	p*	Z	p*	Z	p*	Z	p*	Z	p*
FA_AT_	1.51	0.138	1.19	0.232	2.83	0.005	-3.27	0.001	2.13	0.033	2.27	0.023
FA_startLF_	1.31	0.191	0.95	0.344	2.43	0.015	-2.90	0.004	2.01	0.045	2.33	0.020
FA_endLF_	0.05	0.959	-0.42	0.674	1.66	0.098	-2.21	0.027	2.31	0.021	2.53	0.011
	VD_CC_								VD_CC_			
	Z		p*						Z		p*	
ICGA_AT_	2.05		0.041						0.97		0.335	

*CVR* cardiovascular risk, * p-value after adjusting for age, gender, scan quality index, *Z* Z-statistic, *FA* fluorescein angiography, *ICGA* indocyanine angiography, *VD* vascular density, *SCP* superficial retinal capillary plexus, *DCP* deep retinal capillary plexus, *CC* choriocapillaris, *AT* arterial time, *startLF* start of laminar flow, *endLF* end of laminar flow

### Inter-group comparisons ($\rho$) “CVR” versus “no_CVR”

The vascular densities VD_SCP_, VD_DCP_ were more strongly correlated with the circulatory times FA_AT_, FA_startLF_ and FA_endLF_ in the “no_CVR” group than in the “CVR” group but the differences in strength of the correlation coefficients were non-significant (*p* > 0.05, respectively). On the other hand, vascular density VD_CC_ was significantly more strongly correlated with circulatory time ICGA_AT_ in the “no_CVR” group than in the “CVR” group (*p* = 0.047), Table 2.

### Intra-group “CVR” (ρ) and intra-group “no_CVR” comparisons (ρ)

The vascular density VD_DCP_, compared to the vascular density VD_SCP_, was significantly more strongly correlated with circulatory times FA_AT_, FA_startLF_ and FA_endLF_ (*p* < 0.05, respectively, and with a trend towards significance for FA_endLF_ in the no_CVR group), Table 2. In the “CVR” group, the vascular densities most strongly correlated with circulatory times were significantly observed in diabetic microangiopathy (*p* < 0.05, respectively), Table 2.

### Regression analyses (R.^2^)

**Fig. 2:**
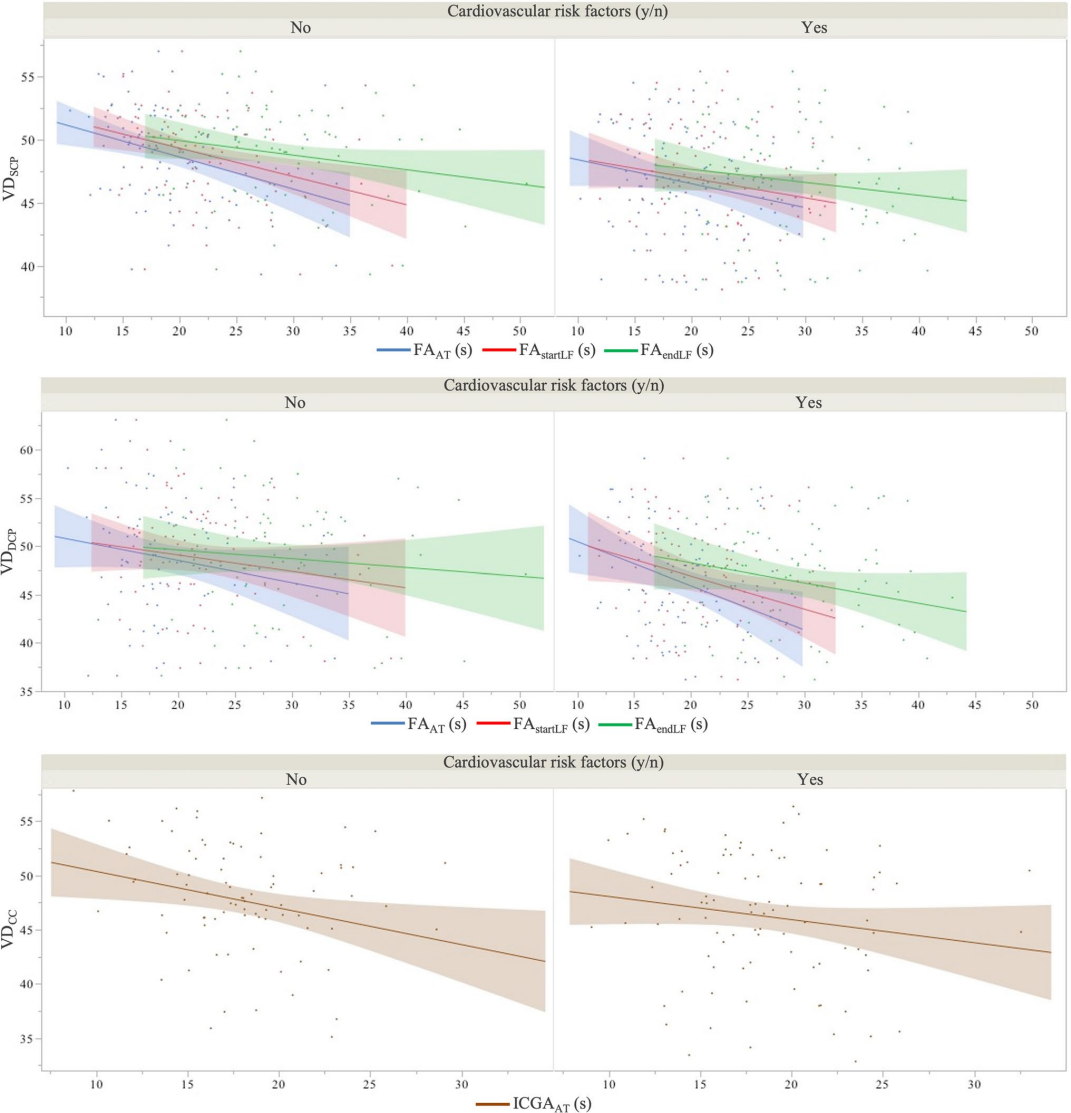
Trendlines of vascular density of capillary plexuses, resulting from the linear regression models implemented from circulatory time data, according to cardiovascular risk

In the overall population and in the “CVR" and “no_CVR” patient groups, the circulatory times FA_AT_, FA_startLF_ and FA_endLF_ had a significant influence on the vascular densities VD_SCP_ and VD_DCP_, likewise ICGA_AT_ on VD_CC_ (*p* < 0.001, respectively), Table 3, Fig. 2. Comparisons of determination coefficients R^2^ of regression models showed that the circulatory times FA_AT_ and FA_startLF_ had a significant greater impact on the vascular densities than the circulatory times FA_endLF_ and ICGA_AT_ (*p* < 0.05, respectively). There was no significant difference in value of the determination coefficients R^2^ between the impacts of circulatory times FA_AT_ and FA_startLF_ on vascular densities (*p* > 0.05 respectively), Table 4.

**Table 3:** Regression analyses assessing the influence of pre-ocular and ocular circulatory dynamics on vascular density of retinal and choroidal capillary plexuses, in patients with and without cardiovascular risk factors

	Without CVR factors				With CVR factors												Cohort			
					All				Diabetes				Other microangiopathies							
*Regression analysis (R*^*2*^*, p-value)*																				
Circulatory times (CTs)	Vascular densities				Vascular densities				Vascular densities				Vascular densities				Vascular densities			
	VD_SCP_		VD_DCP_		VD_SCP_		VD_DCP_		VD_SCP_		VD_DCP_		VD_SCP_		VD_DCP_		VD_SCP_		VD_DCP_	
	R^2^	p*	R^2^	p*	R^2^	p*	R^2^	p*	R^2^	p*	R^2^	p*	R^2^	p*	R^2^	p*	R^2^	p**	R^2^	p**
FA_AT_	*0.715*	< *0.001*	*0.871*	< *0.001*	*0.623*	< *0.001*	*0.833*	< *0.001*	*0.827*	< *0.001*	*0.940*	< *0.001*	*0.566*	< *0.001*	*0.798*	< *0.001*	*0.688*	< *0.001*	*0.859*	< *0.001*
FA_startLF_	*0.660*	< *0.001*	*0.828*	< *0.001*	*0.562*	< *0.001*	*0.785*	< *0.001*	*0.801*	< *0.001*	*0.923*	< *0.001*	*0.496*	< *0.001*	*0.738*	< *0.001*	*0.630*	< *0.001*	*0.814*	< *0.001*
FA_endLF_	*0.404*	< *0.001*	*0.598*	< *0.001*	*0.314*	< *0.001*	*0.549*	< *0.001*	*0.683*	< *0.001*	*0.836*	< *0.001*	*0.229*	< *0.001*	*0.466*	< *0.001*	*0.373*	< *0.001*	*0.579*	< *0.001*
	VD_CC_				VD_CC_				VD_CC_				VD_CC_				VD_CC_			
	R^2^		p*		R^2^		p*		R^2^		p*		R^2^		p*		R^2^		p**	
ICGA_AT_	*0.423*		< *0.001*		*0.262*		< *0.001*		0.624		< *0.001*		0.364		< *0.001*		*0.375*		< *0.001*	

*CVR* cardiovascular risk, * p-value after adjusting for age, gender, scan quality index, ** p-value after adjusting for age, gender, scan quality index, CVR factors, *R*^*2*^ determination coefficient, *FA* fluorescein angiography, *ICGA* indocyanine angiography, *VD* vascular density, *SCP* superficial retinal capillary plexus, *DCP* deep retinal capillary plexus, *CC* choriocapillaris, *AT* arterial time, *startLF* start of laminar flow, *endLF* end of laminar flow

**Table 4:** Comparisons of the value of determination coefficients R^2^ of the impact of pre-ocular and ocular circulatory dynamics on vascular density of capillary plexuses, according to A) circulatory times, B) cardiovascular risk status, C) superficial and deep retinal capillary plexuses and D) diabetic microangiopathy compared to other microangiopathies, in patients with and without cardiovascular risk factors

Comparisons of determination coefficients R^2^of regression models	A) R^2^(CTj, VD) vs R^2^(CT_j’_, VD)												
Circulatory times (CTs)	Without CVR factors						With CVR factors						
	Vascular densities						Vascular densities						
	VD_SCP_			VD_DCP_			VD_SCP_			VD_DCP_			
	Z	p*		Z	p*		Z	p*		Z		p*	
_j_ = FA_AT_ and _j’_ = FA_startLF_	0.669	0.503		0.996	0.3200		0.628	0.530		0.931		0.352	
_j_ = FA_startLF_ and _j’_ = FA_endLF_	2.333	0.020		1.989	0.047		2.073	0.038		2.944		0.003	
	VD_CC_						VD_CC_						
_j_ = FA_startLF_ and _j’_ = ICGA_AT_	2.187	0.029		4.677	< 0.001		2.452	0.014		5.270		< 0.001	
Comparisons of determination coefficients R^2^of regression models	B) R^2^(CT, VD)_no_CVR_ vs R^2^(CT, VD)_CVR_				C) R^2^(CT, VD_SCP_) vs R^2^(CT, VD_DCP_)				D) R^2^(CT, VD)_CVR-diabetes_ vs R^2^(CT, VD)_CVR-others_				
Circulatory times (CTs)	Vascular densities				CVR status				Vascular densities				
	VD_SCP_		VD_DCP_		Without CVR factors		With CVR factors		VD_SCP_		VD_DCP_		
	Z	p*	Z	p*	Z	p*	Z	p*	Z	p*	Z		p*
FA_AT_	1.094	0.274	0.910	0.363	-2.817	0.005	-3.122	0.002	1.890	0.059	2.270		0.023
FA_startLF_	1.026	0.305	0.807	0.420	-2.490	0.013	-2.819	0.005	1.962	0.049	2.333		0.020
FA_endLF_	0.676	0.499	0.477	0.633	-1.675	0.094	-1.948	0.051	2.117	0.034	2.473		0.013
	VD_CC_								VD_CC_				
	Z		p*						Z		p*		
ICGA_AT_	1.196		0.232						0.825		0.410		

*CVR* cardiovascular risk, * p-value after adjusting for age, gender, scan quality index, *Z* Z-statistic, *FA* fluorescein angiography, *ICGA* indocyanine angiography, *VD* vascular density, *SCP* superficial retinal capillary plexus, *DCP* deep retinal capillary plexus, *CC* choriocapillaris, *AT* arterial time, *startLF* start of laminar flow, *endLF* end of laminar flow

Circulatory times had a significant greater impact on vascular densities in the “no_CVR” group than in the “CVR” group but the differences in value of the coefficients of determination R^2^ were non-significant (*p* > 0.05, respectively), Table 4.

Circulatory times FA_AT_, FA_startLF_ and FA_endLF_ had a significantly greater impact on vascular density VD_DCP_ than VD_SCP_, (*p* < 0.05, respectively), Table 4. In the “CVR” group, the greatest impact of circulatory times on vascular densities was significantly observed in diabetic microangiopathy (*p* < 0.05, respectively), Table 4.

The effect of circulatory times on vascular densities was relatively similar in both the full analysis set with missing data imputation and the set of complete-case analysis in which FA_AT_ and FA_startLF_ had the strongest influence on vascular density VD_DCP_ (*p* < *0.001; R*^*2*^ > *0.80,* respectively).

## Discussion

This study investigated the relationships between preocular and ocular vascular circulatory dynamics and vascular density of retinal and choroidal vascular plexuses. The results showed that vascular density of superficial and deep retinal capillary plexuses and choriocapillaris, measured using OCT angiography, was significantly inversely correlated with the circulatory times from fluorescein and indocyanine angiography. The strength of correlations observed between vascular densities of retinal capillary plexuses and circulatory times was not significantly different between patients with and without cardiovascular risk factors. On the other hand, there was a significant linear decorrelation between vascular density of choriocapillaris and circulatory times in patients with cardiovascular risk factors. Some studies showed that the choriocapillaris flow features may follow a power law distribution with implications for mechanisms of disease progression [18]. Furthermore, vascular density of deep retinal capillary plexus was significantly the most strongly correlated with circulatory times, and specifically in diabetic microangiopathy in patients with cardiovascular risk factors. The implemented regression models showed significant changes in vascular density of the retinal and choroidal vascular plexuses under the influence of ocular and preocular circulatory dynamics. Specifically, these analyses demonstrated a significant decrease in vascular density of superficial and deep retinal capillary plexuses as well as choriocapillaris under the influence of lengthening of circulatory times, especially arterial time and start laminar flow time measured by fluorescein angiography, without significant differences between patients with and without cardiovascular risk factors. Vascular density of deep retinal capillary plexus was significantly the most strongly impacted by circulatory times, and specifically in the diabetic microangiopathy in patients with cardiovascular risk factors.

Thus, the results showed that the lengthening of circulatory times statistically influenced the decrease in vascular density of capillary plexuses. However, this did not mean that the lengthening of circulatory times was the cause of the decrease in vascular density of the capillary plexuses. Indeed, influence relationships resulting from regression analyzes do not mean cause-and-effect relationships between the variables circulatory time and vascular density. In addition, the informational value contained in these variables is complex, especially since they can be dependent on other variables or unlisted potential confounding factors. Indeed, various confounding factors, such as age, gender, systemic, cardiovascular diseases and cardiovascular risk factors, medications such as vasodilators or medications that affect kidney or liver function, genetic factors, hydration status and lifestyle such as smoking, diet and exercise can affect circulatory times and retinal and choroidal vascular density by modifying blood circulation. Additional prospective studies, using probabilistic Rothman's approach [19] and its alternatives to explore in some detail the issue of assessing the potential presence of synergism or antagonism in data generated from cohort studies might refine the understanding of circulatory dynamics' impact on capillary vascular density. Deep learning neural networks-based clinical prediction models using these multimodal ophthalmological angiographic imaging data could contribute, more appropriately than multivariate regression models, to bring out the underlying key pathophysiological processes involved in retinal microangiopathies related to CVR factors. Indeed, the results of our study showed a strong multicollinearity between dye-based angiography and OCT angiography variables. This could then compromise the reliability and interpretability of the results of multivariate linear regression models since strong multicollinearity between the variables can generate an inflation of the variance and standard error of estimates of regression coefficients.

Exploration of retinal and choroidal circulatory dynamics involves kinetic analysis of fluorescein and indocyanine molecules, administered intravenously, acting as tracers on angiograms. The kinetics of these tracers depend on their molecular and luminescence characteristics as well as the structural features of retinal and choroidal vasculature. Sodium fluorescein, a molecule of low molecular weight of 376 kDa, predominantly remains intravascular in the retinal capillaries due to the properties of the inner blood-retinal barrier [20] through which neither bound (70% to serum proteins) nor free fluorescein molecules can pass. In the choriocapillaris, unbound molecules escape into the extravascular choroidal space through the choriocapillaris wall fenestrations to the outer blood retinal barrier [20] of the retinal pigment epithelium. While indocyanine green, a molecule of 775 kDa molecular weight approximately 98% bound to serum proteins, is mostly retained within both retinal capillaries and choriocapillaris [21]. Peak fluorescein luminescence is 520–530 nm in the yellow–green light of spectrum, and that of indocyanine, 830–835 nm in the near-infrared (invisible). Infrared (invisible) light enables better penetration of ocular pigments and less scattering than visible light. The differing molecular and luminescence characteristics of fluorescein and indocyanine enable distinct assessments of retinal and choroidal circulation, respectively. The angiogram comprises successive overlapping phases, including pre-arterial (choroidal), arterial, capillary (early and late arteriovenous), and venous phases, enabling the measurement of filling times across these distinct vessels. It presents an overlay of various capillary layers [4, 22–25], primarily showcasing the superficial vascular plexus and the choroidal signal, with limited visualization of the deep capillary plexuses [4, 8, 26].

OCT angiography identifies three distinct macular retinal capillary plexuses: the superficial capillary plexus in the ganglion cell layer, the intermediate capillary plexus in the inner plexiform layer and the internal edge of the inner nuclear layer and the deep capillary plexus in the outer plexiform layer and the external edge of the inner nuclear layer [8, 22, 23, 25, 27]. The superficial and deep capillary plexuses exhibit distinct structural and functional characteristics, potentially accounting for variations in flow resistance and perfusion [22, 28] which may have implications in retinal vascular diseases. The superficial capillary plexus comprises interconnected transverse capillaries linking arterioles and venules [22, 25]. Conversely, the deep capillary plexus is organized into polygonal units, in which the capillaries converge radially toward a central capillary vortex. These capillary vortex centers align along the path of macular superficial venules, and drain into superficial venules through interconnected vertical anastomoses between the superficial and deep capillary plexuses [8, 22, 24, 28, 29]. Choriocapillaris is array into a mosaic of independent polygonal segments, each supplied at its center by a terminal choroidal arteriole and drained by venules arranged on the periphery of the segments, without anastomosis with adjacent segments [30]. Imaging the choriocapillaris in vivo remains challenging due to light scattering within the overlying tissue, especially the retinal pigment epithelium [30]. Therefore, choriocapillaris density should be interpreted critically [30, 31]. Our findings align with the recent studies [22, 25] that designed three-dimensional model depicting certain structural and functional characteristics of retinal capillaries in which venous drainage for the entire retinal microvasculature occurs essentially at the deep capillary plexus level via anastomotic capillary bridges directly connecting the three plexuses. Deep capillary plexus, consisted of capillary units centered around collecting vortex venule, drains directly into the superficial vessels.

The robustness of our study lies in the sample size, as well as quantities and dye injection speeds standardized to ensure the consistency and reproducibility of dye-based angiography times. Nevertheless, our study may have some potential limitations related to selection biases inherent in all retrospective study designs, influence relationships resulting from regression analyses which do not constitute causality relationships, non-automated although protocolized measurements of circulatory times in dye angiography, unsharp demarcation in determining the end of laminar flow in fluorescein angiography, as well as to the potential confounders, such as refractive error although minimized by the inclusion of patients with refractive error limited to ± 3.0 diopters, or the fasting plasma glucose [32] and hematocrit [33] unlisted, and errors in segmentation and measurements of vascular density dependent on the OCT angiogram quality index [34]. However, the statistics were systematically performed after adjustment for angiogram quality index. In addition, only OCT angiograms with a quality index greater than 6 were included in the study to minimize motion, projection, or segmentation artifacts.

## Conclusion

The study highlighted how pre-ocular and ocular circulatory dynamics significantly affect vascular density in the retinal capillary plexuses and choriocapillaris, especially in deep retinal capillary plexuses, relevant for clinical research. Additional prospective studies might refine the understanding of circulatory dynamics' precise impact on capillary vascular density.

## Data Availability

All data relevant to the study are mentioned in this article. Further inquiries can be directed to the corresponding author.

## Abbreviations

CVR: Cardiovascular risks
FA: Fluorescein angiography
ICGA: Indocyanine green near-infrared fluorescence angiography
OCT angiography: Optical coherence tomography angiography
SD: Standard deviation
